# Effect of positive psychological intervention on the treatment and prognosis of patients with acute cerebral infarction

**DOI:** 10.12669/pjms.40.9.7731

**Published:** 2024-10

**Authors:** Yunhe Xu, Xiaojun Li, Xiaoli Guo, Wenbin Gao

**Affiliations:** 1 Yunhe Xu, Key Laboratory of Mental Health, Institute of Psychology, Chinese Academy of Sciences, Beijing 100101, China. University of Chinese Academy of Sciences, Beijing 100049, China; 2 Xiaojun Li, Department of Emergency, Baoding No.1 Central Hospital, Baoding 071000, Hebei, China; 3 Xiaoli Guo, Department of Medical, Baoding No.1 Central Hospital, Baoding 071000, Hebei, China; 4 Wenbin Gao, Key Laboratory of Mental Health, Institute of Psychology, Chinese Academy of Sciences, Beijing 100101, China. University of Chinese Academy of Sciences, Beijing 100049, China

**Keywords:** Acute cerebral infarction, Positive psychological intervention, Prognosis

## Abstract

**Objective:**

To analyze the psychological status of patients with acute cerebral infarction (ACI), and to evaluate the effect of positive psychological intervention on the treatment and prognosis of ACI.

**Methods:**

This was retrospective study. Eighty patients with ACI admitted to Institute of Psychology, Chinese Academy of Sciences from January 2021 to September 2022 were included and randomly divided into observation group (n=40) and control group(n=40). Patients in the control group received conventional treatment and routine care, while those in the observation group received positive psychological intervention based on the control group. Adverse psychological scores, treatment and prognosis, and quality of life as well as nursing satisfaction etc. were analyzed and compared between the two groups.

**Results:**

Both groups showed a significant decrease in SDS and SAS scores at three months after the intervention compared with the pre-intervention period, with a statistically significant difference(P<0.05). After the intervention, the NIHSS score of both groups decreased, with a statistically significant difference(P<0.05). The FMAS and MBI scores increased in both groups compared with those before the intervention, with a statistically significant difference(P<0.05). The SS-QOL scores of both groups were significantly improved compared with before the intervention, with a statistically significant difference(P<0.05). The hospital satisfaction rate in the observation group was significantly higher than that in the control group, with a statistically significant difference (t=12.325, P=0.000).

**Conclusion:**

Positive psychological intervention offers a variety of benefits in the treatment of patients with ACI, such as may alleviate anxiety and depression, reduce neurological deficits, improving quality of life and motor function, and ameliorate the prognosis of patients.

## INTRODUCTION

Nowadays, stroke (including ischemic and hemorrhagic phenotypes) has turned into a common cerebrovascular disease in neurology and emergency departments in hospitals. It remains the primary cause of adult disability, the second leading cause of death globally and the first consistent fatal disease in China.^1^,^2^ With the characteristics of acute onset, severe illness, high incidence and high disability rate, it has been on the rise in recent years in terms of incidence and tends to develop at an increasingly younger age. Stroke makes inroads on approximately 800,000 people each year in the United States. In China, the number of stroke patients reached 13 million in 2017,^3^ and this number is estimated to reach 31.77 million by 2030.^4^ Acute cerebral infarction (ACI), the most common and major type of stroke, is caused by narrowing or complete occlusion of blood vessels due to various causes, resulting in inadequate local blood supply to brain tissue and eventually leading to ischemic and hypoxic necrosis of brain tissue. Although the current significant improvement in medical care has contributed to a significant reduction in the mortality rate of ACI, it still has a disability rate of 40%-60%.^5^ During the long and tortuous process of seeking medical care and treatment, ACI patients and their family members are physically and mentally exhausted, either because of the expensive treatment and rehabilitation costs or the heavy care pressure, which has a serious impact on patients’ psychological and emotional well-being. As a result, patients are prone to anxiety and depression and even have suicidal tendencies, which is not conducive to the recovery and rehabilitation of their disease.^5^ These highlight the still serious situation in the prevention and treatment of ACI.

Researchers have identified a strong correlation between the psychological emotions of patients with ACI and the onset, progression and prognosis of the disease. However, medical staff in clinical practice pay insufficient attention to the psychological aspects of patients. For this reason, it is imperative to find out a psychological intervention model that can effectively adjust patients’ adverse psychological emotions and facilitate their rehabilitation. In this study, the effect of positive psychological intervention on the treatment and prognosis of patients with ACI was primarily evaluated.

## METHODS

This was retrospective study. Eighty patients admitted to Baoding No.1 Central Hospital from January 2021 to September 2022 were recruited as subjects and divided into two groups by random number method: the observation group (treated by positive psychological interventions) and the control group (not treated by positive psychological intervention), with 40 cases in each group.

### Ethical Approval:

This study was approved by the Ethics Committee of Baoding No.1 Central Hospital (No.: [2022]027; date: April 25, 2022), and all patients and their families were informed and consented to participate in this study.

### Inclusion criteria:

- Patients meeting the diagnostic criteria of ACI^6^;
- Patients with stable vital signs, clear consciousness, and no cognitive function or communication disorders;
- Patients with complete relevant data;
- Patients without other severe diseases.

### Exclusion criteria

- Patients with mental diseases and expression disorders;
- Patients with severe heart or liver or kidney disease;
- Patients with poor compliance;
- Patients with coma or cognitive dysfunction;
- Patients with malignant organic diseases.
- Patients with severe abnormalities in coagulation function.

Patients in the control group received conventional intervention on the basis of routine treatment. They were closely monitored for changes in vital signs, provided basic intervention guidance such as life and medication according to the condition, and given corresponding rehabilitation guidance and regular follow-up.The follow-up time for patients in both groups was 6 months.

Based on this, patients in the observation group were given positive psychological intervention on top of the control group. The specific methods are as follows:

- Positive psychological intervention.
- First, effective communication was carried out with patients and their families in a timely manner to evaluate patients’ own advantages for self-management of the disease. Patients were analyzed and evaluated by personnel who had undergone professional psychological knowledge training and assessment, and then hospitalized under humanistic care to alleviate their psychological states such as despair, anxiety, depression and/or fear; Individualized psychological plans were formulated for different patients to help them alleviate their adverse psychology so that they could actively cooperate with disease treatment and rehabilitation.
- Active communication between doctors/nurses and patients was carried out. With humanization as the core basis, high-quality psychological guidance was provided to patients based on their actual conditions, and targeted rehabilitation guidance was carried out. In the rehabilitation period, psychological guidance for patients was strengthened to improve their confidence in life and urge them to develop good living habits.
- Patients were asked to recall friends and relatives who had helped them and were instructed to make grateful return visits to them and to record the complete perception of their state of mind at that time. They were also instructed to recall pleasant daily events related to their illness, and encouraged to vent out their recent unpleasant events and instructed on how to face them properly.
- Patients were asked to record in writing the meaningful events related to ACI before going to sleep every day.

### Health education:

- Health lectures: Health lectures were given in 3-5 sessions to explain the knowledge about ACI, its risk factors and clinical symptoms, diagnosis and treatment methods, as well as the importance of adopting a healthy mindset and lifestyle.
- Written health education: Health education manuals were edited with illustrations to facilitate patients’ understanding, and their main contents were basically the same as the health lectures;
- Distance health education: ACI-related knowledge was shared through the use of WeChat groups, and patients were promptly asked if they had any questions, and medical staff would give timely answers.

### Observation indexes:

- The Self-rating Depression Scale (SDS) was employed to assess patients’ depression status. A total of 20 items are set on this scale with a critical score of 53, with higher scores indicating more severe depressive symptoms. In addition, the Self-rating Anxiety Scale (SAS) was utilized to assess patients’ anxiety status. A total of 20 items are set on this scale with a critical score of 50, with higher scores indicating more severe anxiety symptoms.
- The National Institute of Health Stroke Scale(NIHSS) was used to assess the patients’ neurological deficits. A total of 11 factors are set in this scale, with scores ranging from 0 to 42, with higher scores indicating more severe neurological deficits.
- The Fugl-Meyer Assessment Scale (FMAS) was used to assess patients’ motor function and the Modified Barthel Index (MBI) was used to assess patients’ ability to perform activities of daily living. Both scales were scored as percentages before and after three months of intervention, respectively, with higher scores suggesting better motor function and daily activity ability of the patients.
- ***Assessment of quality of life and self-care ability:*** Before and after the intervention, the Stroke Specific Quality of Life Scale (SS-QOL) was used to assess the quality of life of patients, so as to find out the quality of life of patients, including language, role, cognitive and physical function. The score is 0-20, with higher scores indicating better quality of life.
- Determination of the effect in terms of prognosis of treatment: markedly effective (improvement of symptoms and signs >85%), effective (improvement of symptoms and signs of 50-85%), ineffective (improvement of symptoms and signs <50%). Overall response rate = (markedly effective + effective)/total number of cases × 100%.
- Hospitalization satisfaction was assessed by a self-made scale, with a total score of 100. The scale was divided into three levels: ≥ 90 points as satisfied; 70-89 points as basically satisfied; < 70 points as not satisfied, among which hospitalization satisfaction = (satisfied + basically satisfied)/total number of cases × 100%. All the questionnaires were given to the participants for investigation, and then collected in about five minutes.

### Statistical Analysis:

All data in this study were statistically analyzed using SPSS22.0 software. Measurement data were expressed as (*χ̅*±*S*), and independent sample t test was used for comparison between the two groups before and after treatment. Enumeration data was expressed as n (%), and c² test was used for comparison between the two groups. P<0.05 indicates a statistically significant difference.

## RESULTS

No significant difference was observed in the comparison of general data between the two groups, which was comparable (Table-I). No statistically significant differences were observed in the SDS and SAS scores of the two groups before the intervention (P>0.05). Both groups showed a significant decrease in SDS and SAS scores at three months after the intervention compared with the pre-intervention period, in which the level of decrease was higher in the observation group than in the control group, with a statistically significant difference (P<0.05) (Table-II). No statistically significant differences were observed in the NIHSS score of the two groups before the intervention (P>0.05). After the intervention, the NIHSS score of both groups decreased, with a more significant decrease in the observation group than the control group, with a statistically significant difference (P<0.05) (Table-III).

**Table-I:** Comparison of general data between the two groups.

Item	Observation group (n=40)	Control group (n=40)	t/c^2^	P
Age (years)	56.58±10.73	56.18±9.06	0.180	0.857
Gender (male/female)	25/15	23/17	0.208	0.648
Interval from onset to admission (h)	13.55±2.29	13.43±2.10	0.255	0.800
GCS score			0.453	0.797
9-11 points	15 (37.50%)	13 (32.50%)		
12-14 points	19 (47.50%)	22 (55.00%)		
15 points	6 (15.00%)	5 (12.50%)		

**Table-II:** Comparison of the psychological status of the two groups before and after the intervention (*χ̅*±*S*).

Group	SDS		SAS	

	Before intervention	After intervention	Before intervention	After intervention
Observation group (n=40)	56.98±1.39	36.03±2.81	57.18±1.43	35.78±2.39
Control group (n=40)	57.30±1.47	42.75±2.31	57.55±1.60	40.83±1.89
*t* value	1.017	11.710	1.105	10.472
*P* value	0.312	0.000	0.273	0.000

**Table-III:** Comparative analysis of the neurological deficits between the two groups before and after the intervention (*χ̅*±*S*).

Group	NIHSS	

	Before intervention	After intervention
Observation group (n=40)	21.93±3.50	7.35±1.10
Control group (n=40)	21.88±2.07	8.05±0.60
*t* value	0.078	3.540
*P* value	0.938	0.001

No statistically significant differences were observed in the FMAS and MBI scores of the two groups before the intervention (P>0.05). The FMAS and MBI scores increased in both groups compared with those before the intervention, and the degree of increase was significantly higher in the observation group than in the control group, with a statistically significant difference (P<0.05) (Table-IV).

**Table-IV:** Comparison of motor function and daily living ability of the two groups before and after the intervention (*χ̅*±*S*).

Group	FMAS		MBI	

	Before intervention	After intervention	Before intervention	After intervention
Observation group (n=40)	26.55±1.65	68.95±3.15	35.75±1.21	68.23±3.40
Control group (n=40)	26.43±2.54	66.80±4.39	35.38±1.31	64.70±3.63
*t* value	0.261	2.515	1.325	4.481
*P* value	0.795	0.014	0.189	0.000

No statistically significant differences were observed in the SS-QOL scores of the two groups before the intervention (P>0.05) and these were significantly improved compared with before the intervention, and the degree of improvement of the observation group was significantly better than the control group, with a statistically significant difference (P<0.05) (Table-V). The overall response and overall hospital satisfaction of the observation group was higher than that of the control group, with a statistically significant difference (P<0.05) (Table-VI).

**Table-V:** Comparison of SS-QOL scores between the two groups before and after treatment (*χ̅*±*S*).

Group	Somatic function		Language function		Role function		Cognitive function	

	Before intervention	After intervention	Before intervention	After intervention	Before intervention	After intervention	Before intervention	After intervention
Observation group	4.75±0.44	10.95±0.90	5.90±0.71	15.03±1.10	5.68±0.53	15.58±0.93	7.73±0.55	18.38±0.98
Control group	4.58±0.71	7.50±0.96	5.63± 0.90	11.58±1.24	5.58±0.68	11.40±1.06	7.65±0.66	14.38±1.17
*t* value	1.323	16.537	1.521	13.189	0.739	18.746	0.549	16.584
*P* value	0.190	0.000	0.132	0.000	0.462	0.000	0.584	0.000

**Table-VI:** Comparison of the effectiveness of treatment and prognosis between the two groups [cases (%)].

Group	Markedly effective	Effective	Ineffective	Overall response rate
Observation group (n=40)	27 (67.50)	12 (30.00)	1 (2.50)	39 (97.50)
Control group (n=40)	20 (50.00)	14 (35.00)	6 (15.00)	32 (85.00)
c^2^ value				3.914
*P* value				0.048

## DISCUSSION

The results of this study showed that after intervention, the NIHSS score of the observation group was significantly lower than that of the control group (P<0.05), while the FMAS and MBI scores were significantly higher than those of the control group (P<0.05); After intervention, the scores of physical functions, psychological function, material life, and social function in the observation group were significantly higher than those in the control group (P<0.05). The treatment and outcome effects in the observation group were significantly better than those in the control group (P<0.05), and the satisfaction of hospitalization in the observation group was higher than that in the control group (t=12.325, P=0.000).

It was shown in this study that the reduction level of SDS and SAS scores in the observation group was significantly higher than that in the control group after intervention (P<0.05), indicated that positive psychological intervention can improve negative emotions in patients with ACI. ACI, also known as ischemic stroke, is a disorder of blood circulation to brain tissue that triggers ischemia and hypoxia in brain tissue. It leads to limited ischemic softening or necrosis of brain tissue and accounts for about 80% of strokes.^7^-^9^ Nowadays, medical technology is getting better and better to minimize disability and mortality rates and improve the survival rate of patients with ACI.^10^,^11^ Patients who suffer from the disease experience a stress response that activates the hypothalamic-pituitary-adrenocortical axis and increases the secretion and release of glucocorticoids, leading to a disturbance in the emotional state of patients. As a result, negative psychological emotions such as anxiety and depression are induced, and patients’ healthy behaviors are affected accordingly.^12^-^14^ For most patients with ACI, ACI has a great impact on their physical and mental state, resulting in negative emotions such as fear, anxiety and depression. Once these negative emotions occur, patients may show depression, loss of confidence in recovery and life, or even suicidal tendencies, lack of cooperation with medical staff during treatment, and resistance to rehabilitation exercises. This in turn indirectly or directly affects the treatment and prognosis of the disease.^15^,^16^ For this reason, psychological interventions, especially positive psychological interventions, should be carried out to reduce patients’ psychological stress reactions, help them establish good treatment beliefs, improve clinical cooperation, enhance the treatment and rehabilitation effects of ACI, and improve their quality of life. In addition, patients are able to tap their positive energy in a positive atmosphere, mobilize their social level, promote physical and mental relaxation, and ameliorate their spiritual-psychological condition.^17^

Health education in addition to positive psychological intervention is beneficial for patients to fully understand the occurrence, development, treatment and prognosis of the disease. In addition, patients can also actively cooperate with treatment and rehabilitation, assist to improve the treatment and prognosis of patients, reduce neurological function injury, improve related functions of the body and improve the quality of life of patients.^18^ This indicates that positive psychological interventions can help patients to explore their positive character qualities, mobilize their own strengths to cope with the disease, improve their negative mental condition, face the current situation with a positive attitude, rebuild their confidence in overcoming the disease, motivate individuals to take the initiative to implement various rehabilitation exercises, significantly improve neurological deficits, improve motor function and activities of daily living, and significantly improve patients’ quality of life and self-care ability. This also suggests that positive psychological interventions for ACI patients can significantly prompt inpatient treatment and improve patient satisfaction. The reason for this may be that the positive psychological intervention can make patients realize that they have multiple psychological and physical support, which leads to a good doctor-patient and nurse-patient relationship and improves patients’ adjustment to the disease, thus making them more satisfied during their hospitalization.

### Limitations:

It includes small sample size and short follow-up time. which need to be continued in the future with large samples and long periods of in-depth studies. To address this, more spatients need to be included in future studies and longer in-depth studies should be conducted.

## CONCLUSIONS

Positive psychological intervention results in a variety of benefits in the treatment of patients with ACI, such as it may alleviate anxiety and depression, reduce neurological deficits, promote the formation of healthy behaviors, and produce a positive impact on improving the treatment and prognosis of ACI.

### Authors’ Contributions:

**YX** and **XL:** Carried out the studies, participated in collecting data, and drafted the manuscript, and are responsible and accountable for the accuracy or integrity of the work.

**XG:** Performed the statistical analysis and participated in its design.

**WG:** Participated in acquisition, analysis, interpretation of data and draft the manuscript.

All authors read and approved the final manuscript.
